# 

**DOI:** 10.1192/bjb.2022.89

**Published:** 2023-12

**Authors:** Saloni Peatfield-Bakhshi

**Affiliations:** is a Year 6 Specialty Trainee (ST6) in intellectual disability psychiatry with Birmingham Community Healthcare NHS Foundation Trust, Birmingham, UK. Email: s.peatfield-bakhshi@nhs.net

**Figure:**
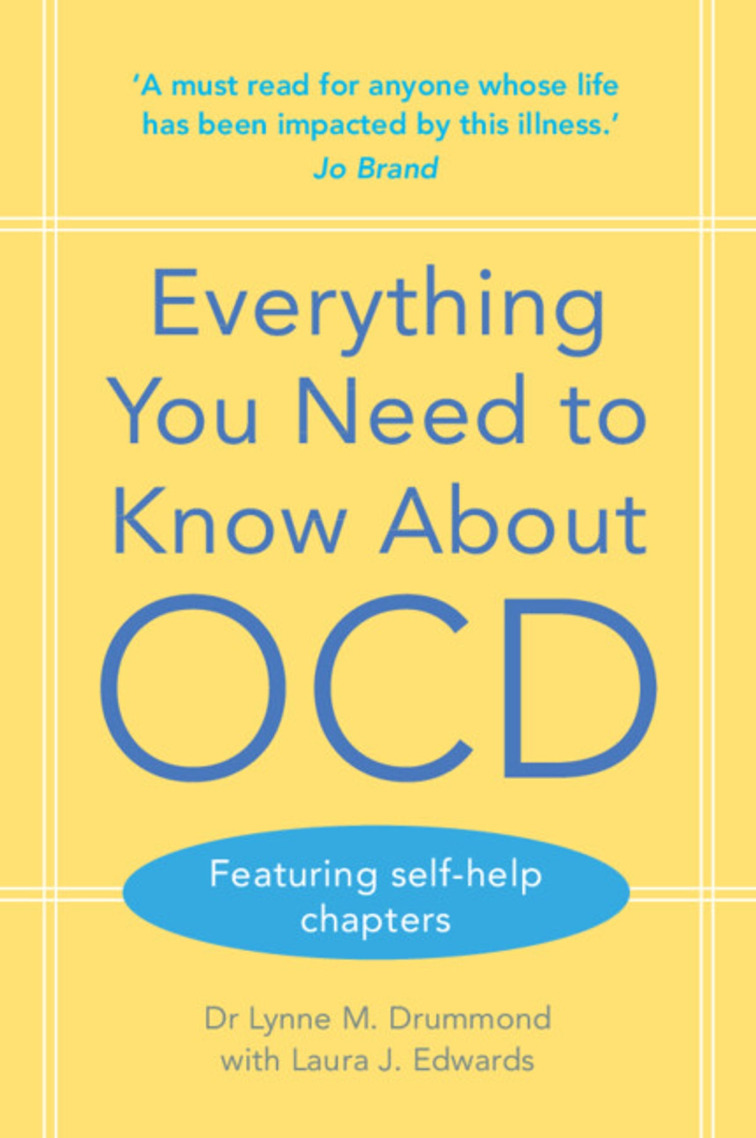

*Everything You Need to Know about OCD* builds on Dr Lynne Drummond's 2018 award-winning book *Obsessive Compulsive Disorders: All You Want to Know about OCD for People Living with OCD, Carers, and Clinicians*. This latest publication focuses on treatment and how to organise a successful treatment programme. Each chapter begins with a useful description of what is to be covered. The book is brought to life by personalised examples of patients’ stories which help to illustrate key points by offering detailed vivid descriptions of the issues affecting the individuals concerned. The personal stories provide a common thread throughout the book, as at times Drummond refers back to previous chapters, recapping the stories and adding another layer to them, thus helping the reader to understand key issues. Each chapter ends with a ‘Key points’ box summarising the important points.

Chapter 4 focuses on drug treatment and provides a useful insight into drugs commonly used to treat OCD, as well as a brief history of drug treatment. Drummond also discusses questions regarding treatment if patients are pregnant and whether medication can alter personalities, as well as whether drug treatments are safe and what to do if they do not work. These are important questions to address as they are often asked by patients. Chapter 7 is an interesting section taking the reader from historical treatments for OCD through to modern developments and new research, finishing with a section on potential treatments for the future. She helps to dispel myths regarding brain surgery and takes the reader through a variety of treatments with a skilful approach making it easy for all to follow.

The self-help chapters in the second half of the book provide useful, practical suggestions for patients, along with helpful links to guidelines. There is also a chapter on how family and carers can help a person with OCD which offers some quite direct and forthright ideas.

A particular strength of this book is its wide-reaching scope in terms of readership. It is suitable for any healthcare practitioner or student regardless of their background, as well as lay people such as individuals with OCD and their families and carers.

